# Host specificity, molecular phylogeny and morphological differences of *Phyllodistomum pseudofolium* Nybelin, 1926 and *Phyllodistomum angulatum* Linstow, 1907 (Trematoda: Gorgoderidae) with notes on Eurasian ruffe as final host for *Phyllodistomum* spp.

**DOI:** 10.1186/s13071-017-2210-9

**Published:** 2017-06-06

**Authors:** Virmantas Stunžėnas, Romualda Petkevičiūtė, Larisa G. Poddubnaya, Gražina Stanevičiūtė, Alexander E. Zhokhov

**Affiliations:** 1 Nature Research Centre, Akademijos str. 2, LT-08412 Vilnius, Lithuania; 2 I.D. Papanin Institute for Biology of Inland Waters, Russian Academy of Sciences, 152742 Borok, Yaroslavl Province Russia

**Keywords:** *Phyllodistomum pseudofolium*, *P. angulatum*, Eurasian ruffe, Life-cycles, ITS2 rDNA, 28S, Host specificity, SEM, Morphological variation

## Abstract

**Background:**

Host-specificity patterns are not well-defined for trematodes of the genus *Phyllodistomum* Braun, 1899. The Eurasian ruffe, *Gymnocephalus cernuus* L., has been recorded as a definitive host for *Phyllodistomum folium* (Olfers, 1816), *P. angulatum* Linstow, 1907 and *P. megalorchis* Nybelin, 1926 and as the type-host for *P. pseudofolium* Nybelin (1926). A wide range of other host fishes have been recorded for these species as well. All present host records have been based on light microscopy and the life-cycles of *P. pseudofolium*, *P. angulatum* and *P. megalorchis* are unknown. The validity of *P. pseudofolium* and *P. megalorchis* require verification. In this study, rDNA sequences generated from adult *Phyllodistomum* spp., as well as from larval stages developing in *Pisidium amnicum* Müller, were analysed to establish the real number of *Phyllodistomum* species utilizing *G. cernuus*, and to associate larvae with the corresponding adult forms.

**Results:**

Phylogenetic analyses of adult and larval stages of *Phyllodistomum* spp. based on ITS2 and partial 28S rDNA data allowed the confirmation of the validity of *P. pseudofolium*. A macrocercous cercaria, known as *Phyllodistomum* sp. from *P. amnicum* is genetically identical to adult *P. pseudofolium. Phyllodistomum megalorchis* obtained from its type-host, *Lota lota* L., showed no genetic differences from *P. angulatum* parasitizing *Sander lucioperca* L*.* In our analysis, *P. pseudofolium*, *P. angulatum* and *P. macrocotyle* formed a highly supported clade despite the fact that these species appear to be associated with distinct patterns of first intermediate host identity and cercarial morphology. Some morphological differences between gravid specimens of *P. pseudofolium* and *P. angulatum* were observed and their SEM tegumental surface topography is described.

**Conclusions:**

The results lead us to the perception that macroevolutionary host switching in the genus *Phyllodistomum* is independent of host phylogeny*.* This study suggests strict host-specificity (oioxeny) for *P. pseudofolium* using one first intermediate host species (*P. amnicum*) and one definitive host species (*G. cernuus*)*. Phyllodistomum megalorchis* is to be regarded as a synonym of *P. angulatum*. The close phylogenetic relatives, *P. pseudofolium* and *P. angulatum*, can be differentiated by morphological traits, the micromorphology and tegumental surface topography of these two species is intended to provide useful data for their identification and support the use of such features as a valuable taxonomic criterion. Molecular data showed that *G. cernuus* is a definitive host for two species: the oioxenous *P. pseudofolium* and the euryxenous *P. folium.*

## Background

Host specificity is arguably one of the most important properties of parasitic organisms. Several definitive and intermediate hosts can be involved in helminth life-cycles, thereby complicating the pattern of specificity. Molecular analysis has often shown that species of parasites once thought to be generalists (euryxenic or stenoxenic) were, in reality, complexes of specialist (oioxenic) species generally recognized as cryptic species (see [1, 2]). As a result, generalist species parasites are considered with suspicion.

The digenean genus *Phyllodistomum* Braun, 1899 (Gorgoderidae) contains around 120 species, which typically inhabit the urinary bladder and/or ureters of both marine and freshwater fishes, more rarely amphibians [3–10]. Taxonomic confusion in the genus *Phyllodistomum* is caused greatly by the absence of a well-defined host specificity pattern. Moreover considerable intraspecific variability has been found in most morphological and morphometric features of these digeneans [3, 11–14].

According to literature, the Eurasian ruffe *Gymnocephalus cernuus* L. (Percidae) has been recorded as a definitive host for five species of the genus *Phyllodistomum*: *P. pseudofolium* Nybelin (1926), *P. angulatum* Linstow, 1907, *P. megalorchis* Nybelin, 1926, *P. simile* Nybelin, 1926 and *P. folium* (Olfers, 1816) [15, 16]. Based on these data, *G. cernuus* should be one of the fish host species harboring the greatest variety of *Phyllodistomum* spp. Each one of the *Phyllodistomum* species listed above has a long and complicated taxonomic history.

Nybelin [17] studied parasites from ureters of *G. cernuus* collected in Sweden and, on the basis of comparative analysis of his findings and the works of Looss [18], Lühe [19] and Odhner [20], erected a new species, *P. pseudofolium*. According to Bykhovskaya-Pavlovskaya & Kulakova [21], this parasite may infect other definitive hosts, mostly zander, *Sander lucioperca* L., and perch, *Perca fluviatilis* L*.* The validity of this species has been accepted by some helminthologists [21, 22] while rejected by others [3, 23] and is still questionable; its life-cycle is unknown. Pigulevsky [22] noted that the presumed intermediate hosts of *P. pseudofolium* are sphaeriid clams.

*Phyllodistomum angulatum* was described by Linstow in 1907 [24] based on material from *S. lucioperca* caught in the River Volga. Later it was found in other fish hosts of the families Percidae, Esocidae and Cyprinidae [*Sander volgensis* (Gmelin), *P. fluviatilis*, *Esox lucius* L., *Leuciscus idus* L.,* Alburnus alburnus* L.], but rarely in *G. cernuus* (see [21, 22]). The species has yet to be reported in Lithuania [25].

*Phyllodistomum megalorchis* was recorded in *G. cernuus* in Latvia by Kirjušina & Vismanis [26]. Originally, the species was described from *Lota lota* L., *Salmo trutta* L., *Thymallus thymallus* L. and *Phoxinus phoxinus* L. Dawes [23] considered that *P. megalorchis* is synonymous with *P. simile*, and *P. simile*, in turn, “is likely to prove identical with *P. folium”*. Comparative molecular analysis proved the identity and, consequently, synonymy of *P. simile* and *P. folium* [27].

The type-species of the genus *Phyllodistomum*, *P. folium* was described by Olfers [28] based on specimens recovered in *E. lucius*. However, the description was deficient. Later, Looss [18] presented both a description and figure of the specimens of *Phyllodistomum* from Eurasian ruffe (*G. cernuus* L.; as *Acerina cernua*) identified as *P. folium*, which replaced Olfers’s [28] original and was later used as *P. folium* in many survey publications [19, 23, 29]. Pigulevsky [22] stated that *P. folium* is a specific parasite of *E. lucius*, an opinion supported by Moravec [30] who found *P. folium* exclusively in *E. lucius*. We confirmed the identity of *P. folium* using molecular markers; its host specificity appeared the lowest among the known *Phyllodistomum* spp.: adults of *P. folium* were detected in eight teleost species from five families and four orders, including *E. lucius* and *G. cernuus* [27]. Cystocercous cercariae of *P. folium* were recorded in sphaeriid bivalves of the genus *Sphaerium* Scopoli and *Pisidium* Pfeiffer [27].

Elucidation of life-cycles is critical to a complete understanding of gorgoderid trematodes, but the vast majority of gorgoderid life-cycles remain unknown. Cercariae produced in the life-cycles of *Phyllodistomum* spp. include different types, indicating a diversity not necessarily reflected by the morphology of adults [31, 32]. The most common type of life-cycle described is that characterized by cystocercous cercariae, but rhopalocercous and a microcercous cercaria are also known as larval stages of phyllodistomes [5]. Some yet unassociated gorgoderid cercariae, presumably attributable to the genus *Phyllodistomum*, have been described from freshwater bivalves in Europe. One of them, cercaria *Phyllodistomum* sp. *sensu* Ginetzinskaya, 1959 [33] was described from *Pisidium amnicum* (Müller, 1774) collected in the Rybinsk Water Reservoir (estuary of the River Volga, Russia) [33]. This cercaria has a long tail not enclosing the cercarial body and a short stylet embedded in the oral sucker. Ginetzinskaya [33] thought, based only on morphology, that this cercaria is the larva of *P. angulatum.* In previous studies, a match was not detected between cercaria *Phyllodistomum* sp. *sensu* Ginetzinskaya, 1959 and any adult *Phyllodistomum,* including *P. angulatum* [27].

While morphology may not be enough to establish robust species delimitation criteria, scanning electron microscopy (SEM) studies have revealed distinct patterns of the distribution of papillae on the body surfaces of species of the family Gorgoderidae [34–37], including species of *Phyllodistomum* [8–10, 38–43]. These studies have suggested that the arrangements of these tegumental papillae on adult gorgoderid species represent useful taxonomic characters.

This study is the first attempt to genetically characterize *P. pseudofolium* to test the species validity, as well as to study its phylogenetic affinities, host range and specificity. Ribosomal DNA sequences generated from adult stages of *Phyllodistomum* spp. collected from Eurasian ruffe *G. cernuus* and other fish species, as well as generated from larval stages developing in *P. amnicum* were compared and analyzed to establish the true number of *Phyllodistomum* species utilizing *G. cernuus* as a definitive host and to associate larvae with the corresponding adult form.

## Methods

Larval and adult gorgoderids were collected from bivalves and from urinary bladders and ureters of freshwater fishes in different water bodies in Lithuania and Russia (Table 1). Total genomic DNA for molecular analysis was isolated according to protocols of Stunžėnas et al. [44] with a slight modification described in Petkevičiūtė et al. [45]. DNA fragments spanning the 3’ end of 5.8S rRNA gene, complete internal transcribed spacer 2 region (ITS2) and a small section at the 5' end of the 28S gene were amplified using the forward primer 3S (5'-CGG TGG ATC ACT CGG CTC GTG-3') [46] and the reverse primer ITS2.2 (5'-CCT GGT TAG TTT CTT TTC CTC CGC-3') [47] that anneal to the beginning of the large subunit (28S) near the ITS2. A fragment at the 5' end of the 28S rRNA gene was amplified using forward primer Digl2 (5'-AAG CAT ATC ACT AAG CGG-3') and reverse primer L0 (5'-GCT ATC CTG AG (AG) GAA ACT TCG-3') [48]. Amplification protocols are as described in Petkevičiūtė et al. [45]. PCR products were purified and sequenced in both directions at BaseClear B.V. (Leiden, the Netherlands) using PCR primers. Contiguous sequences were assembled using Sequencher 4.7 software (Gene Codes Corporation). New sequences of *P. pseudofolium*, *P. angulatum*, *P. folium* and *Phyllodistomum* sp. have been deposited in the GenBank (see accession numbers in Table 1).

**Table 1:** Species used in molecular phylogenetic analysis with information of their host, locality and GenBank accession numbers

Species	Host	Locality	GenBank ID [Reference]	
			28S	5.8S-ITS2-28S
*Cercaria duplicata* ^a^	*Anodonta anatina*	Lake Saravesi, Finland	KJ729516 [27]	KJ740490 [27]
*C. duplicata* ^a^	*A. anatina*	Kaunas Water Reservoir, Lithuania	KJ729515 [27]	KJ740492 [27]
*C. duplicata* ^a^	*A. anatina*	River Chesnava, Russia	KJ729514 [27]	KJ740491 [27]
*C. duplicata* ^a^	*A. anatina*	River Sluch, Ukraine	KJ729517 [27]	KJ740489 [27]
*Phyllodistomum folium*	*Esox lucius*	River Ild, Russia	KJ729542, KJ729545 [27]	KJ740499, KJ740500 [27]
*P. folium*	*Rutilus rutilus*	Rybinsk Water Reservoir on River Volga, Russia	KJ729536 [27]	KJ740501 [27]
*P. folium*	*Aspius aspius*	Rybinsk Water Reservoir on River Volga, Russia	KJ729548 [27]	KJ740503 [27]
*P. folium*	*Abramis ballerus*	Rybinsk Water Reservoir on River Volga, Russia	KJ729539 [27]	KJ740502 [27]
*P. folium*	*Abramis brama*	Rybinsk Water Reservoir on River Volga, Russia	KJ729537, KJ729547 [27]	KJ740505 [27]
*P. folium*	*Gymnocephalus cernuus*	River Shumorovka, Russia	KJ729552 [27]	KJ740506 [27]
*P. folium* ^b^	*G. cernuus*	River Chesnava near Rybinsk Water Reservoir on River Volga, Russia	KX957727, KX957728	KY307880–KY307883
*P. folium* ^b^	*G. cernuus*	Curonian Lagoon, Lithuania	KX957729	KY307884, KY307885
*P. folium*	*Cottus gobio*	River Nėris, Lithuania	KJ729550 [27]	KJ740507 [27]
*P. folium*	*Gasterosteus aculeatus*	River Vilnelė, Lithuania	AY277707 [62]	AY277705 [62]
*P. folium* ^a^	*Sphaerium corneum*	River Shumorovka, Russia	KJ729546 [27]	KJ740498 [27]
*P. folium* ^a^	*S. corneum*	River Hegga, Norway	KJ729549, KJ729551 [27]	KJ740494, KJ740495 [27]
*P. folium* ^a^	*Pisidium supinum*	River Ūla, Lithuania	KJ729544 [27]	KJ740496 [27]
*P. folium* ^a^	*Pisidium amnicum*	River Loo Jõgi, Estonia	KJ729541 [27]	
*P. folium* ^a^	*P. amnicum*	River Ild, Russia	KJ729535 [27]	KJ740497 [27]
*P. folium* ^a^	*P. amnicum*	River Siilaisenpuro, Finland	KJ729533 [27]	
*Phyllodistomum umblae*	*Coregonus albula*	Lake Syamozero, Karelia, Russia	KJ729528 [27]	KJ740508, KJ740509, KJ740510 [27]
*Phyllodistomum angulatum*	*Sander lucioperca*	River Chesnava, Russia	KJ729529, KJ729530, KJ729531 [27]	KJ740511, KJ740512 [27]
*P. angulatum* ^b^	*S. lucioperca*	Rybinsk Water Reservoir on River Volga, Russia	KX957734, KX957735	
*P. angulatum* ^b^ (*= Phyllodistomum megalorchis*)	*Lota lota*	Rybinsk Water Reservoir on River Volga, Russia	KX957733	KY307870, KY307871
*Phyllodistomum pseudofolium* ^b^	*G. cernuus*	River Chesnava near Rybinsk Water Reservoir on River Volga, Russia	KX957727, KX957731, KX957732	KY307875, KY307876–KY307879
*P. pseudofolium* ^a^ (= *Phyllodistomum* sp*.* of Ginetzinskaya, 1959)	*P. amnicum*	River Chesnava, Russia	KJ729527 [27]	KJ740513 [27]
*P. pseudofolium* ^ab^ (= *Phyllodistomum* sp*.* of Ginetzinskaya, 1959)	*P. amnicum*	Pond near Rybinsk Water Reservoir on River Volga, Russia	KX957730	KY307874
*Phyllodistomum* sp.^b^ (identified as *P. pseudofolium*)	*Perca fluviatilis*	Rybinsk Water Reservoir on River Volga, Russia	KY307869	KY307886
*Phyllodistomum macrocotyle* ^a^	*Dreissena polymorpha*	Lake Vilkokšnis, Lithuania	KJ729518, KJ729522, KJ729526 [27]	KJ740518, KJ740519 [27]
*P. macrocotyle* ^a^	*D. polymorpha*	Elektrėnai Water Reservoir, Lithuania	KJ729525 [27]	KJ740516 [27]
*P. macrocotyle* ^a^	*D. polymorpha*	Lake Wigry, Poland	KJ729520 [27]	KJ740520, KJ740521, KJ740522 [27]
*P. macrocotyle* ^a^	*D. polymorpha*	River Shumorovka, Russia	KJ729521, KJ729524 [27]	KJ740523 [27]
*P. macrocotyle* ^a^ (*= P. folium sensu* Sinitsin, 1905)	*D. polymorpha*	Lake Lepelskoe, Belarus	AY288828 [63]	AY288831 [63]
*P. macrocotyle* ^a^ (*= P. folium sensu* Sinitsin, 1905)	*D. polymorpha*	Lake Lukomskoe, Belarus	AF533015 [63]	AF533015 [63]
*P. magnificum*	*Tandanus tandanus*	Moggill Creek, Queensland, Australia	KF013186, KF013189 [53]	KF013153, KF013156 [53]
*P. inecoli*	*Heterandria bimaculata*	Agua Bendita, Xico, Veracruz, Mexico	KC760199 [8]	
*P. inecoli*	*Profundulus* sp.	Rio Templo, San Juan del Rio, Mexico	KM659389 [10]	
*P. spinopapillatum*	*Profundulus balsanus*	Rio PuebloViejo, San Gabriel Mixtepec and Rio Santa Cruz, Santiago Jamiltepec, Mexico	KM659382, KM659388 [10]	
*P. centropomi*	*Centropomus parallelus*	Tlacotalpan, Veracruz, Mexico	KM659384 [10]	
*P. kanae*	*Hynobius retardatus*	Pippu, Hokkaido, Japan	AB979868 [7]	
*P. brevicecum*	*Umbra limi*	Brokenhead, Manitoba, Canada	KC760204 [8]	
*P.* cf*. symmetrorchis*	*Clarias gariepinus*	Lake Victoria, Kenya	KF013174 [53]	KF013162 [53]
*P. vaili*	*Mulloidichthys flavolineatus*	Lizard Island, Queensland, Australia	KF013173 [53]	KF013155 [53]
*P. hoggettae*	*Plectropomus leopardus*	Lizard Island, Queensland, Australia	KF013191 [53]	KF013148 [53]
*Pseudophyllodistomum johnstoni* ^a^	*Macrobrachium australiense*	Warrill Creek, Queensland, Australia	KF013177 [53]	KF013166 [53]
*Gorgodera cygnoides*	*Rana ridibunda*	Switzerland	AF151938 [64]	
*G. cygnoides*	*R. ridibunda*	Kokaljane, near Sofia, Bulgaria	AY222264 [65]	
*G. amplicava*	*R. catesbeiana*	Nebraska, USA		FJ445743 [66]
*Gorgoderina attenuata*	*R. clamitans*	Nebraska, USA		FJ445741 [66]
*G. simplex*	*R. clamitans*	Nebraska, USA		FJ445742 [66]
*Xystretrum solidum*	*Sphoeroides testudineus*	Conch Key, Florida, USA	KF013188 [53]	KF013149 [53]
*Xystretrum* sp.	*Sufflamen fraenatum*	Ningaloo, Western Australia, Australia		KF013160 [53]
*Nagmia floridensis*	*Rhinoptera bonasus*	Gulf of Mexico, East Ship Island, Mississippi, USA	AY222262 [65]	
*Cercariaeum crassum* ^*a*^	*P. amnicum*	River Žeimena, Lithuania	GU462117 [67]	
*Maritrema arenaria* ^a^	*Semibalanus balanoides*	Belfast Lough, Northern Ireland		HM584171 [68]

^a^Sequences generated from larval stages

^b^Sequences generated in the present study

Additional rDNA sequences of gorgoderid species and outgroup taxa (Table 1) were downloaded from GenBank and included in pairwise sequence comparisons and phylogenetic analyses. For the phylogenetic analyses, both the ITS2 and 28S datasets were aligned using ClustalW [49] with an open gap penalty of 15 and gap extension penalty of 6.66. The best-fit model of sequence evolution for phylogenetic analysis was estimated using jModeltest v. 0.1.1 software [50]. Ambiguously aligned positions were excluded from phylogenetic analysis. Maximum Likelihood (ML) phylogenetic trees were obtained and analyzed using MEGA v6 [51]. Branch support was estimated by bootstrap analyses with 1,000 pseudoreplicates. The ML trees were obtained using the general time reversible model with a gamma distribution rate and a proportion of invariant sites (GTR + G + I) for both the ITS2 and the 28S gene datasets. Gamma shape and the number of invariant sites were estimated from the data. Parsimony analysis based on subtree pruning and regrafting (SPR) was used with default parsimony settings. If two or more sequences belong to one species, they were collapsed into one branch, except those of *P. pseudofolium* and *P. angulatum*. Estimates of mean evolutionary divergence over sequence pairs within and between groups were calculated using the MEGA v6 programme.

Seventeen specimens of *P. pseudofolium* from *G. cernuus*, seventeen specimens of *P. angulatum* from *S. lucioperca* and five specimens of *P. angulatum* from *L. lota* were used for light microscopy examination. All these specimens were adult and gravid. The flukes were placed in 6.5% saline, killed in hot 10% formalin-saline according to the protocol of Bakke [13], stored in 70% ethanol and stained with alum carmine, dehydrated in ascending concentrations of ethanol, cleared in dimethyl phthalate and mounted in Canada balsam. All measurements are in micrometers.

For scanning electron microscopy, live specimens of *P. angulatum* and *P. pseudofolium* were fixed in 3% glutaraldehyde in 0.1 M sodium cacodylate buffer (pH 7.2) for 20 days at 5 °C and then dehydrated in a graded ethanol series, with a final change to absolute acetone. The worms were critical point-dried with liquid CO_2_ and then mounted on stubs, sputter-coated with gold-palladium and examined using a JEOL JSM 6510LV scanning electron microscope operating at 30 kV.

## Results

### General results

Prevalence and intensity of infection with *Phyllodistomum* spp. were different in the studied fish species. Out of the 169 individuals of *G. cernuus* studied, 31% were infected with 1–9 trematodes per fish. The molecular studies identified that 19% of the studied *G. cernuus* were infected with *P. pseudofolium* and 12% with *P. folium*; no mixed infections were detected. All (100%) dissected *S. lucioperca* (17 fishes) were infected with *P. angulatum* with an intensity of 53–243 trematodes per fish. A total of 87 individuals of *L. lota* were dissected, but only 3 fishes (3.5%) were infected with 1–3 *Phyllodistomum* spp. specimens per fish. *Perca fluviatilis* infection with *Phyllodistomum* was rare in the studied water bodies and only one gravid specimen was recovered from *P. fluviatilis.*

### Phylogenetic analysis

Sequence data of two different regions of rDNA (ITS2 region and partial 28S gene) of adult *Phyllodistomum* spp. from *G. cernuus*, *S. lucioperca*, *L. lota* and *P. fluviatilis* were compared. All adult *Phyllodistomum* specimens from *G. cernuus*, preliminary identified as *P. angulatum*, and partenitae of *Phyllodistomum* sp. *sensu* Ginetzinskaya, 1959 from *P. amnicum*, were genetically identical to *P. pseudofolium* from its type-host *G. cernuus*. All other adult *Phyllodistomum* spp. specimens from *G. cernuus* were genetically identical to *P. folium*. Comparison of the rDNA sequences confirmed the morphological identification of *Phyllodistomum* specimens from *S. lucioperca* as *P. angulatum*. However, *P. megalorchis* from *L. lota* appeared genetically identical to *P. angulatum*. 28S rDNA sequence for *Phyllodistomum* sp. from *P. fluviatilis* was different from those for *P. pseudofolium* and *P. angulatum*, and more similar to the sequences for *P. folium* and *P. umblae*.

**Fig. 1:**
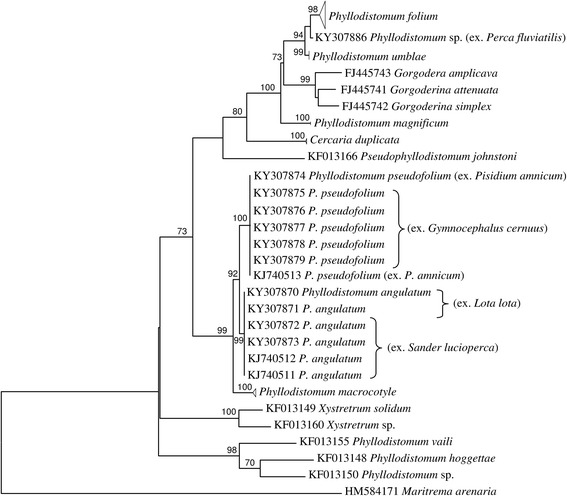
Phylogenetic tree based on Maximum Likelihood analysis of the ITS2 nuclear rDNA region. Bootstrap support values lower than 70% are not shown. GenBank accession numbers of the collapsed clades are provided in Table 1

**Fig. 2:**
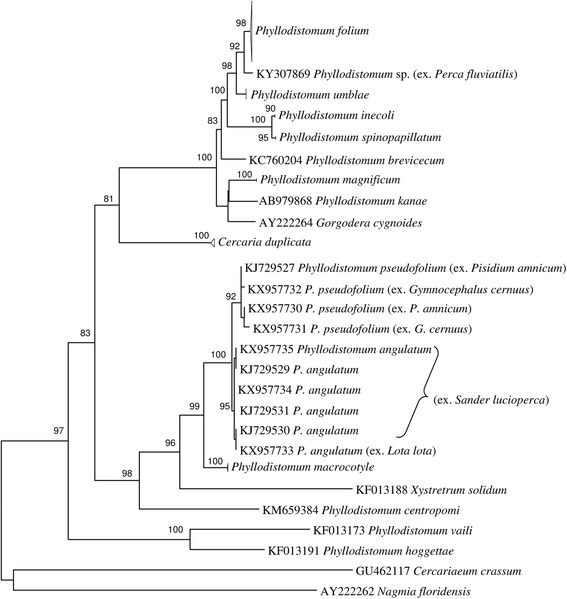
Phylogenetic tree based on Maximum Likelihood analysis of partial sequences of the 28S nuclear rDNA gene. Bootstrap support values lower than 70% are not shown. GenBank accession numbers of the collapsed clades are provided in Table 1

Alignment of the ITS2 rDNA and 28S rDNA regions for gorgoderid taxa yielded 418 and 1,094 characters for phylogenetic analysis, respectively. Phylogenetic analyses of these two datasets produced similar groupings into several strongly supported clades (Figs. 1, 2). Sequences of adult specimens of *P. pseudofolium* together with partenitae of *Phyllodistomum* sp. *sensu* Ginetzinskaya, 1959 and adult *P. angulatum* clustered in two sister subclades. They formed a highly supported clade together with *P. macrocotyle* (Figs. 1, 2). *Phyllodistomum* sp. from perch, identified as *P. pseudofolium*, but molecularly different, clustered in the 28S tree between *P. folium* and *P. umblae* clades (Figs. 1, 2).

### Morphological differences based on light microscopy

**Fig. 3:**
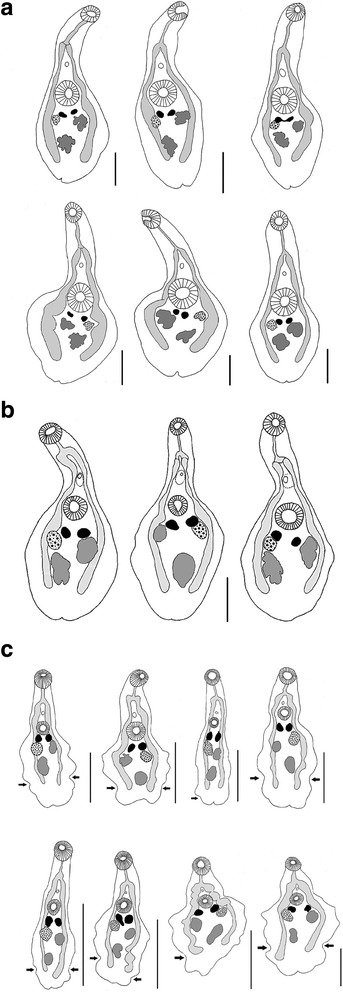
Morphological variability in species of *Phyllodistomum*. **a** *P. angulatum* Linstow, 1907 ex *Sander lucioperca*, **b** *P. angulatum* ex *Lota lota,* **c** *P. pseudofolium* Nybelin, 1926 ex *Gymnocephalus cernuus*, arrows show contractions of hindbodies at the level of caecal terminations. *Scale-bars*: 500 μm

Body shape was found to be influenced by the way the specimens are killed and fixed. The lateral margins of the hindbody of *P. angulatum* from *S. lucioperca* and *L. lota* remain smooth (without undulations and lateral flaps) after fixation in hot 10% formalin-saline (Fig. 3a, b). The mid-ventral lateral flaps, a typical diagnostic character for *P. angulatum*, are preserved only in cold fixation (room temperature). The hindbody of *P. angulatum* is oval, round or rhomboid in shape. The oral sucker is smaller than the ventral sucker (Table 2). *Phyllodistomum pseudofolium* has two to three undulations on each lateral side of the hindbody. The last contraction of the hindbody is always situated at the level of caecal terminations (Fig. 3c). The oral and ventral suckers are similar in size (Table 2). Gravid *P. pseudofolium* differs from *P. angulatum* in having smaller and more rounded eggs (Table 2). Additional significant morphological differences were not detected between these species.

**Table 2:** Measurements (in μm) for the main morphological features showing differences between gravid specimens of *P. pseudofolium* and *P. angulatum*

Parasite	*P. pseudofolium* *(n* = 17)	*P. angulatum* *(n* = 17)	*P. angulatum* *(n* = 5)
Host	*Gymnocephalus cernuus*	*Sander lucioperca*	*Lota lota*
Body length	1,007–1,764 (1,290.6)	2,304–2,988 (2,560)	2,736–3,744 (3,168)
Body width	315–1,080 (567)	720–1,476 (1,127)	1,224–1,656 (1,462)
Oral sucker width	138–252 (175)	207–297 (253)	243–387 (310)
Ventral sucker width	126–216 (171)	306–450 (392)	360–513 (430)
Sucker ratio	0.85–1.27 (1.03)	0.56–0.74 (0.65)	0.68–0.77 (0.72)
Eggs (*n* = 20)	29–33 × 16–20 (30 × 19)	33–40 × 18–20 (35 × 20)	33–40 × 20–22 (37 × 21)

### Tegumental topography of *Phyllodistomum angulatum*

**Fig. 4:**
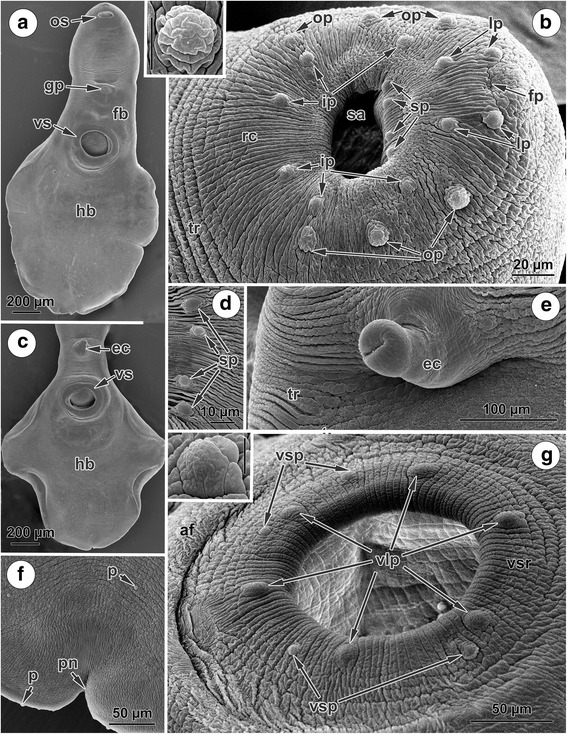
SEM micrographs of the surface topography of *Phyllodistomum angulatum*. **a**, **c** Body of a mature worm, ventral view. **b** Oral sucker rim; note a constant pattern of 20 sensory papillae: four papillae localised on each side of the frontal pit; six papillae arranged in an outer ring; six papillae arranged in an inner ring and four papillae are situated within the sucker cavity (inset, papilla of oral sucker, *scale-bar*: 10 μm). **d** Four papillae within oral sucker cavity. **e** Evaginated cirrus; note smooth tegumental surface around and on evaginated cirrus. **f** Posterior notch, dorsal view. **g** Ventral sucker rim showing six large constant papillae and four small papillae; note negligible ‘acetabular fold’ (inset, papilla of the ventral sucker, *scale-bar*: 10 μm). *Abbreviations*: af, acetabular fold; ec, evaginated cirrus; fb, forebody; fp, frontal pit; gp, genital pore; hb, hindbody; ip, papilla of the inner ring; lp, lateral papilla surrounding frontal pit; op, papilla of the outer ring; os, oral sucker; p, papilla; pn, posterior notch; rc, radial corrugations of the sucker rim; sa, sucker aperture; sp, small papilla within sucker cavity; tr, transverse tegumental ridges; vlp, large papilla of the ventral sucker rim; vs, ventral sucker; vsp, small papilla of the ventral sucker rim

Under SEM, shallow, transverse tegumental ridges are apparent on the ventral surface of both the forebody and hindbody of *P. angulatum* and also along the dorsal side of the body (Fig. 4b, c, e). The rims of the oral sucker exhibit radially oriented corrugations (Fig. 4b). A consistent pattern of sensory papillae occurs around the apertures of the both suckers (Fig. 4b, g). Additionally, a few similar papillae are scattered irregularly on the ventral, lateral and dorsal surfaces of the fore- and hindbody (Fig. 4f).

At the anterior extremity, dorsal to the oral sucker, a rather indistinct frontal pit is present (Fig. 4b). On each lateral side of the frontal pit two papillae are situated about 14 μm from each other; the distance between papillae on opposite sides of the pit is about 24 μm (Fig. 4b). In addition to these four papillae, a further 16 papillae are consistently associated with the oral sucker, 12 of which (6 and 6) are arranged in two rings (outer and inner) on the sucker rim; the distance (about 14 μm) between these rings is the same as between the lateral papillae associated with the frontal pit (Fig. 4b). The distance between the papillae in the inner ring is about 37 μm, and between those in the outer ring (in threes, symmetrically arranged on each side of the sucker) is about 31 μm. Papillae in the same ring have a similar size, but those in the inner ring are smaller than those in the outer ring (Fig. 4b). The remaining four papillae occur antero-dorsally within sucker cavity and are smaller in size to those in the inner ring (Fig. 4b, d).

The rim of the ventral sucker bears six large distinct dome-shaped papillae regularly distributed and arranged in a single ring (Fig. 4g). In addition, four irregularly scattered smaller papillae occur slightly external to this ring in the postero-lateral region of the sucker (Fig. 4g). All these papillae are hidden inside retracted ventral suckers (Fig. 4a, c). A negligible fold, the so-called ‘acetabular fold’ in digeneans (see [52]), is present around the ventral sucker primarily posteriorly (Fig. 4g).

The cirrus was observed projecting from the genital pore, which is situated medially on the ventral surface of the forebody between the two suckers (Fig. 4c, e). In contrast to the ventral surface, the surface of the genital atrium and the evaginated cirrus is smooth and lacks papillae, readily distinguishing it from the surrounding body tegument (Fig. 4f).

A notch at the posterior extremity of the body, within which the excretory pore is located, is equally visible in both dorsal and ventral views (Fig. 4f). Close to the excretory pore, papillae occur along the lateral surface of the posterior region of the body (Fig. 4f).

### Tegumental topography of *Phyllodistomum pseudofolium*

**Fig. 5:**
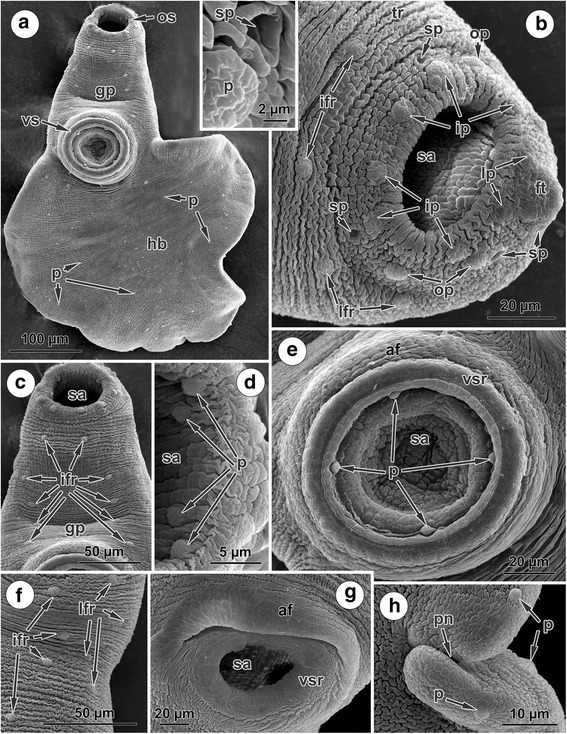
SEM micrographs of the surface topography of *Phyllodistomum pseudofolium*. **a** Body of a mature worm, ventral view. **b** Oral sucker rim; note two papillae situated on each side of the frontal tubercle, six papillae arranged in an inner ring, two lateral pairs of papillae, and four papillae situated within the sucker cavity (inset, papilla and secretory pore close to the oral sucker). **c** Forebody, ventral view showing five pairs of papillae arranged symmetrically in two ventro-lateral rows. **d** Four symmetrically arranged papillae on the internal surface of the oral sucker. **e** Partly retracted ventral sucker showing four symmetrically arranged papillae within the sucker cavity; note rather negligible ‘acetabular fold’. **f** Part of the ventral surface of the forebody showing the presence on each side of both ventro-lateral and lateral rows of papillae. **g** Retracted ventral sucker showing the lack of papillae on the sucker rim and pronounced ‘acetabular fold’. **h** Posterior notch, dorsal view; note the irregular surface and lateral papillae. *Abbreviations*: af, acetabular fold; ft, frontal tubercle; gp, genital pore; hb, hindbody; ifr, inner papilla on the ventral surface of the forebody; ip, papilla of the inner ring of the oral sucker; lfr, papilla of the lateral row on the ventro-lateral margin of the forebody; lp, lateral papilla beside the frontal pit; op, papilla of the outer ring of oral sucker; os, oral sucker; p, papilla; pn, posterior notch; sa, sucker aperture; sp, secretory pore; tr, transverse tegumental ridges; vs, ventral sucker; vsr, ventral sucker rim

Most of the dorsal and ventral surfaces of the adult worms are covered by transverse tegumental ridges (Fig. 5a-c). The rim of the subterminal oral sucker bears radially oriented corrugations (Fig. 5b). A posterior notch is visible in the middle of the postero-dorsal margin of the body, where the surrounding tegument is less irregular (Fig. 5h).

A frontal tubercle is situated dorsal to the anterior margin of the oral sucker as a small, prominent anterior elevation with a ventro-lateral papilla on each side (Fig. 5b). In addition to these two papillae, there is a consistent pattern of six other sensory papillae arranged along the rim of the oral sucker (Fig. 5b); these form the inner ring. Just external to the inner ring is an outer ring of four papillae formed by two symmetrical pairs, one on each side of the sucker (Fig. 5b). Within the oral sucker cavity there are a further four papillae arranged in two symmetrical pairs on the internal antero-lateral wall (Fig. 5b-d). Also surrounding the oral sucker are six secretory pores; these are arranged in three symmetrical pairs on either side of the sucker: one pair postero-lateral to the rim, one pair antero-lateral to the rim and the other pair lateral to the frontal tubercle (Fig. 5b, inset).

Four papillae are present just inside the cavity of the partly retracted ventral sucker (Fig. 5e); all these papillae are hidden inside the retracted ventral sucker (Fig. 5g). The primarily posteriorly ‘acetabular fold’ is more pronounced in the specimens with a retracted ventral sucker (Fig. 5g), around the partly retracted sucker this fold is negligible (Fig. 5e).

The genital pore is situated ventro-medially in the forebody between the two suckers, closer to the ventral sucker (Fig. 5a, c). On each side of the forebody there is a longitudinal, ventro-lateral row of five large papillae (Fig. 5c). In addition, symmetrically on each lateral side of the forebody, eight smallest papillae are arranged in a longitudinal row (lateral row) (Fig. 5f).

There is no distinct arrangement of papillae on the tegument of either the dorsal or ventral sides of the hindbody, although a few papillae are irregularly distributed (Fig. 5a). The posterior margin of the body, in the region of the posterior notch, is papillated (Fig. 5h).

### Taxonomic summary based on new molecular and morphological data

*Phyllodistomum angulatum* Linstow, 1907 (syn. *Phyllodistomum megalorchis* Nybelin, 1926).

*Type-host*: *Sander lucioperca* L. (Percidae).

*Other host*: *Lota lota* L. (Lotidae).

*Type-locality*: River Volga.

*Other localities*: Sweden near Upsala.

*Site in host*: Urinary bladder, ureters.

*Voucher material*: No. 1/15 (1–10) are deposited in the Parasites Collection of the Institute for Biology of Inland Waters RAS, Russia.

*Representative DNA sequences*: KJ729529–KJ729531, KJ740511, KJ740512, KY307870, KY307871, KX957733–KX957735.

*Phyllodistomum pseudofolium* Nybelin, 1926

*Type-host*: *Gymnocephalus cernuus* L.

*Type-locality*: Sweden near Upsala.

*Other localities*: River Volga (Rybinsk Reservoir).

*Site in host*: Urinary bladder.

*Voucher material*: No. 1/14 (1–7) are deposited in the Parasites Collection of the Institute for Biology of Inland Waters RAS, Russia.

*First intermediate host*: *Pisidium amnicum* (Müller, 1774).

*Representative DNA sequences*: KY307875, KY307876–KY307879, KX957727, KX957731, KX957732.

## Discussion

In recent years, there have been considerable advances in our understanding of the systematics and phylogeny of gorgoderid digeneans [8, 27, 53, 54]. Nevertheless, many unanswered questions on species diversity, validity and life-cycles are still waiting clarification. The taxonomy of *P. pseudofolium*, as well as many other species of the genus *Phyllodistomum*, is complicated. Cribb [32] discussed the difficulties of proper identification among *Phyllodistomum* spp. due to the great intraspecific morphological variation in many species and numerous inadequate morphological descriptions. Not only new species descriptions, but also delimitation criteria for known species, will urgently require the use of molecular markers to discriminate species. Nevertheless, preliminary identification and species delimitation inevitably involves morphological criteria. The present data, based on comparative analysis of ITS2 and 28S gene sequences, and on light and SEM microscopy examination, unequivocally support the species validity of *P. pseudofolium*. In all analyses, sequences of *P. pseudofolium* from *G. cernuus* formed a genetic lineage well separated from other *Phyllodistomum* species for which data are available. No match was found between sequences of *Phyllodistomum* sp. from *P. fluviatilis*, identified as *P. pseudofolium* on morphological traits, and other known *Phyllodistomum* spp. In the phylogenetic analysis, this *Phyllodistomum* sp. clustered in one clade with *P. folium* and *P. umblae.* Therefore, *Phyllodistomum* sp. from *P. fluviatilis*, identified as *P. pseudofolium* based on morphology, must be regarded as a new, as yet to be described species. Further studies on its morphology and identity are required. The results of this study suggest strict host specificity (oioxeny) for adult and larval stages of *P. pseudofolium.* Throughout our vast molecularly based studies of the target fishes infected with *Phyllodistomum* digeneans, the molecular identity of *P. pseudofolium* was confirmed only for specimens obtained from one fish species, *G. cernuus*. The larval stages of *P. pseudofolium* were detected only in *P. amnicum.* This trematode is genetically closest to *P. angulatum*, so Ginetzinskaya [33] was not far from the truth when, based only on the morphology of cercaria of *Phyllodistomum* sp. from *P. amnicum*, she presumed that it is a larval stage of *P. angulatum.*

Supposedly, the species richness within the genus *Phyllodistomum* is underestimated, as has been highlighted by the molecular studies by Rosas-Valdez et al. [54] and Peribáñez et al. [55] who demonstrated the likely existence of complexes of cryptic species of *Phyllodistomum* in North America and Europe, respectively. However, our study revealed the opposite, i.e. some European species (e.g. *P. elongatum* Nybelin, 1926 and *P. simile*) appeared to be conspecific with *P. folium* and must be synonymized [27]; consequently the number of valid species was reduced. The results of our study disprove the validity of *P. megalorchis* obtained from its type-host, *L. lota*. The morphology of *P. angulatum* parasitizing in *L. lota* closely resembles the description of *P. megalorchis* and differs from the description of the ‘typical’ *P. angulatum*, most likely due to the influence of the host, as it was presumed by Kudinova [3] who noted that the development of marita (maritogony) and resulting morphology of gravid *Phyllodistomum* specimens is determined by the anatomy of fish urinary system.

Recent molecular phylogenetic studies involving members of the Gorgoderidae have shown that the genus *Phyllodistomum*, which is the most species-rich genus in the family Gorgoderidae and also one of the largest genera in the Digenea, is paraphyletic [8, 27, 53]. Distinct phylogenetic units grouped under *Phyllodistomum* (*sensu lato*) may be characterised by different cercarial morphology. Cutmore et al. [53] suggested that their first intermediate host identity and some aspects of life-cycle biology may be important keys to these clades. Life-cycles of the some gorgoderines are known, particularly for some *Phyllodistomum* species that are parasitic in freshwater fishes in Europe. However, comparative molecular analysis of respective adult and larval forms disproved all life-cycles established by experimental infections or based on ecological evidence (see [27]). It was believed for a long time that *P. folium* utilizes the dreissenid bivalve *Dreissena polymorpha* (Pallas) as the first intermediate host and possesses microcercous cercariae as first reported by Sinitsin [56]. Ivantsiv & Kurandina [57] showed in their experimental study that the rhopalocercariae developing naturally in the clam *Anodonta anatina* L. (= *Anodonta ponderosa* C. Pfeiffer) are cercariae of *P. angulatum*. Experimental infection of *Tinca tinca* L. and *Carassius auratus* L. by Orecchia et al. [58] demonstrated that rhopalocercous *Cercaria duplicata* von Baer, 1827 from *Anodonta cygnaea* L. is the larval form of *P. elongatum*. However, Zhokhov [59] identified the cercaria of *P. elongatum* as a cystocercous cercaria developing in *P. amnicum*. Eventually, molecular and karyological data matched cystocercous cercariae from sphaeriid bivalves with adult *P. folium*; no match was revealed between *C. duplicata* and any species of *Phyllodistomum*, including adults found in experimental studies; molecular results support the conspecificity of *P. elongatum* and *P. folium*. At present, no first intermediate host is known for *P. angulatum* and the adult form of *C. duplicata* remains undiscovered.

Our comparative molecular analysis showed that *P. amnicum*, a freshwater bivalve of the family Sphaeriidae, harbors the parasitic asexual stages of *P. pseudofolium*. The cercaria is similar to other described gorgoderid cercariae. In not surrounding the cercarial body, the tail of this cercaria is similar to the gorgoderid cercariae described by Coil [31, 60] from North American unionid bivalves and to the cercaria of *Pseudophyllodistomum johnstoni* Cribb, 1987 developing in corbiculid bivalves from Australia [32]. In molecular phylogenies, a well-supported clade comprises *P. folium*, *P. umblae* and other gorgoderine species in which cystocercous cercariae develop in sphaeriid bivalves, and such clustering of species with similar life-cycles supports the presumption that distinct phylogenetic units grouped under *Phyllodistomum* (*sensu lato*) may be characterised by different aspects of life-cycle biology. However, in our analysis *P. pseudofolium*, *P. angulatum* and *P. macrocotyle* nested in a highly supported clade despite the fact that *P. pseudofolium* and *P. macrocotyle* appear to be associated with distinct patterns of first intermediate host identity and distinct cercarial morphology. The microcercous cercaria of *P. macrocotyle* uses the dreissenid bivalve *D. polymorpha* as intermediate host. It should be noted, that this cercaria in the general consensus [55] was mistakenly considered as a larva of *P. folium* since the publication of Sinitsin [56]. The recent genetic studies of *P. folium* and *P. macrocotyle* [27] have proven that only *P. macrocotyle* is a parasite of *D. polymorpha* among the phyllodistomes. The long-tailed macrocercous cercaria of *P. pseudofolium* infects the gills of the sphaeriid bivalve *P. amnicum*. Then, host specificity of larval and/or adult *Phyllodistomum* spp*.* appears not directly related to host phylogeny*.* Even phylogenetically closely related species or specimens of one species (for example, *P. folium*, *P. angulatum*) can infect hosts from different families or orders. According to Gibson [11] indications from European *Phyllodistomum* spp. suggest that some degree of host group specificity (stenoxeny) is involved. New species, such as *P. inecoli* Razo-Mendivil, Pérez-Ponce de León & Rubio-Godoy, 2013 described from Mexico [8] are usually described from a single fish species. However additional results incorporating molecular markers revealed more wide specificity, stenoxeny (fish hosts species from different families of the order Cyprinodontiformes) [10]. Euryxeny (fish hosts from three different orders) was revealed for *P. magnificum* Cribb, 1987 from freshwater fishes in Australia and New Zealand [32]. However these data require further molecular verification because molecular markers are available only for specimens from one host species [53]. Such wide host switching during the evolution within the genus *Phyllodistomum* could be determined by ecological factors, historical interactions between the definitive and intermediate hosts or multiple geographical isolations.

*Gymnocephalus cernuus* is native to most European countries and has been introduced to many European waters where it was not native, as well as to the North American Great Lakes. Ogle [61] listed 63 parasites of *G. cernuus*. Based on molecular evidence, two species, *P. elongatum* and *P. megalorchis*, should be regarded as synonyms of *P. folium* and *P. angulatum*, respectively.

Light microscopy and SEM observations provided additional sources of information for species discrimination. Thus, the gravid specimens of *P. pseudofolium* with two to three undulations in each lateral margin of hindbody distinctly differ from *P. angulatum* with oval, round or rhomboid hindbody. The two species also differ in the sucker ratio: the oral and ventral suckers of *P. pseudofolium* are similar in size, while the ventral sucker of *P. angulatum* is larger than the oral sucker.

The SEM observations of adult *P. angulatum* and *P. pseudofolium* revealed the presence of only aciliate sensory papillae. According to Bakke [39, 40], there are four types of sensory papillae on the surface of *P. umblae* [= *P. conostomum* (Olsson, 1876)], which are distinguished by their shape (button-shaped, ciliated, dome-shaped and rosette papillae) and partly by their location. In reality, we can assume, based on our SEM observations, that it is possible to identify only two types of papillae, ciliate and aciliate. The shape of ‘other types’ of papillae may depend on the level of surface invaginations in various *Phyllodistomum* spp., as sensory endings localized within the tegumental syncytial cytoplasm tend to look like surface outgrowths under SEM. The type of sensory papillae can be identified correctly using transmission electron microscopy, since, by using this technique, it is possible to determine the nature of the sensory endings and, hence, their classification. Nevertheless, as in the present SEM study of *P. angulatum* and *P. pseudofolium*, only one type of sensory papillae was reported for *P. folium* by Bakke & Zdarska [41].

The present SEM of the surface topography of *P. angulatum* and *P. pseudofolium* revealed different patterns in the regular arrangement of papillae. Thus, *P. angulatum* is characterized by 20 papillae around the oral sucker (4 on both sides of the frontal pit + 6 in the inner ring on the sucker rim + 6 in the outer ring on the sucker rim + 4 within sucker cavity); the size of the papillae decreases from outer to inner rings with their smaller size within sucker. Yet, in *P. pseudofolium,* 16 uniformly-sized papillae are associated with the oral sucker (2 on both sides of the frontal tubercle + 6 in the inner ring on the sucker rim + 4 in the outer ring on the sucker rim + 4 within sucker cavity). Additionally, 6 secretory pores surround the oral sucker of *P. pseudofolium*. The ventral sucker is a dynamic structure. The non-retracted ventral sucker of *P. angulatum* has six large characteristic papillae and four smaller irregular papillae. On the partly retracted ventral sucker of *P. pseudofolium*, four uniformly-sized papillae were observed, while all these papillae on the ventral sucker are hidden inside the retracted ventral sucker. How many papillae are hidden inside the partly retracted ventral sucker of *P. pseudofolium* could only be established from SEM of non-retracted ventral suckers. Unfortunately, there were no specimens of *P. pseudofolium* examined by SEM where the ventral sucker was not in a retracted position, so additional study is needed here. A few papillae are scattered irregularly on the ventral surface of the forebody of *P. angulatum*; this region of *P. pseudofolium* is characterized by the presence of four symmetrically longitudinal rows of papillae, two ventro-lateral rows of five large papillae and two lateral rows of eight smallest papillae. Finally, a notch at the posterior extremity of the body in *P. angulatum* is equally visible in both dorsal and ventral views, while in *P. pseudofolium* the notch is visible only in dorsal view.

Judging from the present SEM results, the specific arrangement of the papillae found in each of these two species can be used as a basis for the identification of specimens from the urinary system of *S. lucioperca* and *G. cernuus*. On the basis of a comparative analysis of the arrangement of papillae in other species belonging to the genus *Phyllodistomum* which have been studied using the SEM, i.e. *P. umblae* [38–40], *P. folium* [41], *P. funduli* [43], *P. inecoli* [8], *P. cribbi* and *P. wallacei* [9] and *P. spinopapillatum* [10], it is apparent that a specific arrangement occurs on the body surface of each investigated species, which exhibit inter-specific differences in the number, arrangement and type of papillae.

## Conclusions

Recent DNA studies provide a new approach to unravel the taxonomic status of nominal *Phyllodistomum* species with complicated taxonomic history and to clarify their life-cycles. However, it is necessary to collect many more samples from different hosts for molecular studies to evaluate host specificity patterns in *Phyllodistomum* spp. Comparative molecular studies accompanied by morphological analysis of *Phyllodistomum* spp. enable plausible recognizing and delimitation of species. For now, we can state that European *Phyllodistomum* spp. differ greatly in their degree of host specificity. Species comprising well-supported clades in molecular phylogeny do not necessarily follow the same life-history patterns. The new data on the validity, host specificity and life-cycles of phyllodistomes, as well as species-specific markers obtained in this study, will be valuable for phylogenetic revision of the genus *Phyllodistomum*. This study showed that *G. cernuus* is the definitive host for two *Phyllodistomum* species: the oioxenous *P. pseudofolium* and the euryxenous *P. folium.* All other *Phyllodistomum* spp. detected in *G. cernuus* could be the result of incorrect identification by light microscopy and should be revised considering molecular markers.

## Abbreviations

ITS2: Internal transcribed spacer 2
ML: Maximum likelihood
SEM: Scanning electron microscopy
SPR: Subtree pruning and regrafting
